# Supplementary material for: Forward Genetics by Sequencing EMS Variation-Induced Inbred Lines
Source: G3 (Bethesda). 2016 Dec 29;7(2):413–25. doi: 10.1534/g3.116.029660 (PMC5295590; doi:10.1534/g3.116.029660)
Supplement: Supplementary file 1 [file 413TableS1.pdf]

Table S1. Summary of preliminary and filtered G:C to A:T transitions in the whole genome and protein-coding region SNPs in the error-prone SNPs filtering.

Line	WG[a] gcat%	WG N[b]	CDS[c] gcat%	CDS[d] N	-BSWG[e] gcat%	-BSWG[f] N	-BSCDS[g] gcat%	-BSCDS[h] N
10-2	49.70	19,452	45.51	3,032	77.21	10,426	69.16	1,741
10-3	59.60	15,566	59.06	1,876	84.77	9,636	91.07	1,097
10-d	50.43	18,155	44.95	2,605	78.87	9,663	71.95	1,408
12-2	53.94	4,885	57.89	1,033	77.93	2,891	80.84	663
dhr2-1	29.58	13,000	32.19	1,839	65.45	3,184	79.58	524

[a] WG gcat% refers to Percentage of G:C to A:T SNPs in the whole genome prior to filtering.
[b] WG N refers to Number of G:C to A:T SNPs in the whole genome prior to filtering.
[c] CDS gcat% refers to Percentage of G:C to A:T SNPs in the coding sequences prior to filtering.
[d] CDS N refers to Number of G:C to A:T SNPs in the coding sequences prior to filtering.
[e] -BSWG gcat% refers to Percentage of G:C to A:T SNPs in the whole genome after filtering.
[f] -BSWG N refers to Number of G:C to A:T SNPs in the whole genome after filtering.
[g] -BSCDS gcat% refers to Percentage of G:C to A:T SNPs in the coding sequences after filtering.
[h] -BSCDS N refers to Number of G:C to A:T SNPs in the coding sequences after filtering.
